# A Three-Stage Accelerometer Self-Calibration Technique for Space-Stable Inertial Navigation Systems

**DOI:** 10.3390/s18092888

**Published:** 2018-08-31

**Authors:** Qiuping Wu, Ruonan Wu, Fengtian Han, Rong Zhang

**Affiliations:** Department of Precision Instrument, Tsinghua University, Beijing 100084, China; wrn13@mails.tsinghua.edu.cn (R.W.); hft@mail.tsinghua.edu.cn (F.H.); rongzh@mail.tsinghua.edu.cn (R.Z.)

**Keywords:** space-stable INS, accelerometer calibration, system-level calibration, device-level calibration

## Abstract

As a specific force sensor, the tri-axis accelerometer is one of the core instruments in an inertial navigation system (INS). During navigation, its measurement error directly induces constant or alternating navigation errors of the same order of magnitude. Moreover, it also affects the estimation accuracy of gyro drift coefficients during the initial alignment and calibration, which will indirectly result in navigation errors accumulating over time. Calibration can effectively improve measurement accuracy of the accelerometer. Device-level calibration can identify all of the parameters in the error model, and the system-level calibration can accurately estimate part of these parameters. Combining the advantages of both the methods and making full use of the precise angulation of the space-stabilized platform, this paper proposes a three-stage accelerometer self-calibration technique that can be implemented directly in the space-stable INS. The device-level calibration is divided into two steps considering the large amount of parameters. The first step is coarse calibration, which identifies parameters except for the nonlinear terms, and the second step is fine calibration, which not only identifies the nonlinear parameters, but also improves the accuracy of the parameters identified in the first step. The follow-on system-level calibration is carried out on part of the parameters using specific force error and attitude error to further improve the calibration accuracy. Simulation result shows that by using the proposed three-stage calibration technique in the space-stable INS, the estimation accuracy of accelerometer error can reach ${1\times 10}^{-6}\;g$ order of magnitude. Experiment results show that after the three-stage calibration, the accuracy of latitude, longitude, and attitude angles has increased by over 45% and the accuracy of velocity has increased by over 22% during navigation.

## 1. Introduction

Inertial navigation systems (INSs) tend to be the first choice for marine navigation because they are able to output motion parameters completely, autonomously, and continuously [1,2,3]. In order to fulfill the requirement for long-term and high-precision marine navigation, it is essential for INSs to employ high-precision inertial instruments. The gyroscope should be able to work stably for a long time, of which the drift error needs to be small and the constant drift can be precisely compensated. And as for the accelerometer, high stability of the bias and the scale factor is required [4].

All of the inertial instruments must be precisely tested and calibrated before they can work properly to provide accurate measurement in the INS [5,6]. According to the observed quantities, accelerometer calibration can be divided into two types, device-level calibration and system-level calibration. In device-level calibration, the accelerometer is set to measure the gravity of Earth at different positions by using a dividing head, a centrifuge, or a turntable, and error parameters are identified according to the outputs of the accelerometer [7,8,9]. The accelerometer data measured during the multi-position rotation of the turntable are compared with the theoretical value of the local gravity, and then using the least square method, error parameters can be estimated from their difference. The system-level calibration is based on the propagation of error parameters of inertial instruments to the final navigation errors. Error parameters can be precisely identified with a proper rotation scheme that is designed considering the parameter observability [3].

Jiang et al. [9] proposed a device-level calibration method based on the static-base condition, and the precise attitude and attitude rate provided by a tri-axis precision turntable, where the calibration accuracy can reach ${1\times 10}^{-6}\;g$ order of magnitude. However, in practical applications, it is difficult to provide an ideal laboratory environment for the implementation of this method. Won et al. [10] and Ye et al. [11] calibrated the accelerometer on the basis that the amplitude of the output vector of the tri-axis accelerometer equals 1 g. Studies [12,13,14,15,16,17,18,19,20,21] presented several system-level calibration methods specific to the microelectromechanical system (MEMS) inertial measuring unit (IMU), but the calibration accuracy needs to be improved. The methods proposed in references [22,23,24,25,26,27,28,29,30] are appropriate for the rotational INSs, and several novel methods are presented for the hybrid INSs in references [31,32]. Gao et al. [22] used position error and velocity error as the observed quantities to estimate the nonlinear error coefficients. They also used velocity error and attitude error to estimate the error coefficients of the gyroscope and the accelerometer [23]. Pan et al. [33] calibrated the nonlinear term in the scale factor based on the change rate of velocity error. Gao et al. [27] and Liu et al. [31] proposed methods to compensate the size effect and the lever arm effect, respectively. As for the turntable rotation strategy, the 6-position [22], 9-position [24,32], 18-position [18,34], and 24-position [35] methods have been proposed. In existing works, calibration methods are rarely designed for the space-stable INSs. In addition, the complementary benefits of device-level and the system-level calibration have not been taken into consideration.

In the space-stable INS, the platform is stabilized in inertial space by the gimbal servo system. With no applied torque, the moment of momentum of the rotor in the free-rotor gyroscope maintains fixed orientation in inertial space, which can ensure the high precision of the system [1]. As there is a stabilized platform in a space-stable INS, self-calibration based on such an own condition becomes the current development trend. This paper attempts to combine the advantages of full-parameter estimation in the device-level calibration and high-precision estimation of partial parameters in the system-level calibration. A three-stage self-calibration technique is proposed for the high-precision accelerometer calibration in practical applications. Firstly, device-level self-calibration is conducted directly in the system. It is divided into two steps considering the large amount of parameters. The first step is coarse calibration which identifies parameters except for the nonlinear terms, and the second step is fine calibration which identifies the nonlinear terms and simultaneously improves the accuracy of parameters identified in the first step. Secondly, the follow-on system-level calibration is carried out on part of the parameters using the errors of specific force and attitude angles to further improve the calibration accuracy. The proposed method is specific to the space-stable INS and has three contributions. First, taking advantage of the high-precision angulation ability of the gimbaled platform, the two-stage device-level calibration can be directly implemented in the system. Second, based on the error propagation of the residual accelerometer model error to navigation errors, the system-level calibration uses specific force error and attitude error to form the observation model. Third, the proposed three-stage calibration method merges the complementary advantages of both the device-level calibration and the system-level calibration to achieve full-parameter calibration of the accelerometer measurement model as well as high-precision estimation of part of these parameters. This can help further improve the performance of the system.

The rest of this paper is arranged as follows: In Section 2, the accelerometer measurement model is introduced. The device-level coarse calibration and device-level fine calibration are presented in Section 3 and Section 4, respectively. The system-level calibration method based on the errors of specific force and attitude angles is proposed in Section 5. Simulation and experiment results are discussed in Section 6. And finally conclusions are drawn in Section 7.

## 2. Accelerometer Measurement Model

For the convenience of following discussion, several coordinate frames are defined first, and the superscript of a vector will indicate which frame it is in.

$O_{n}x_{n}y_{n}z_{n}$—the local geographic navigation frame (*n*-frame), where the $x_{n}$ axis and the $y_{n}$ axis are in the local-level plane pointing north and east, respectively, and the $z_{n}$ axis is angled vertically downward. The gravity vector ***g*** has the same orientation with the $z_{n}$ axis in this coordinate system.

$O_{b}x_{b}y_{b}z_{b}$—the body-fixed coordinate frame (*b*-frame). The origin $O_{b}$ is usually located at the center of the mass of the vehicle. The $x_{b}$ axis points forward along the longitudinal axis, the $z_{b}$ axis points to the bottom, and the $y_{b}$ axis points rightward to form a right-handed coordinate system.

$O_{p}x_{p}y_{p}z_{p}$—the platform-fixed coordinate system (*p*-frame). The $z_{p}$ axis is along the axis of revolution of the platform. And ideally, $x_{p}$ and $y_{p}$ are along the revolution axes of the inner gimbal and the middle gimbal, respectively.

$O_{a}x_{a}y_{a}z_{a}$—the accelerometer coordinate system (*a*-frame), which is fixed on the tri-axis accelerometer. The $x_{a}$, $y_{a}$ and $z_{a}$ axes are along the three sensitive axes, respectively. This coordinate system is non-orthogonal due to the installation error.

For a static system, the specific force measured in the *n*-frame should be:

(1) $$f^{n}={\left[\begin{array}{ccc} 0 & 0 & -g \end{array}\right]}^{T},$$

where *g* is the scalar gravity. Transforming it to the *p*-frame yields:

(2) $$f^{p}=C_{b}^{p}C_{n}^{b}f^{n},$$

where the direction cosine matrix from the *n*-frame to the *b*-frame is:

(3) $$C_{n}^{b}=\left[\begin{array}{ccc} \cos\psi \cos\theta & \sin\psi \cos\theta & -\sin\theta \\ -\sin\psi \cos\phi +\cos\psi \sin\theta \sin\phi & \cos\psi \cos\phi +\sin\psi \sin\theta \sin\phi & \cos\theta \sin\phi \\ \sin\psi \sin\phi +\cos\psi \sin\theta \cos\phi & -\cos\psi \sin\phi +\sin\psi \sin\theta \cos\phi & \cos\theta \cos\phi \end{array}\right],$$

and the direction cosine matrix from the *b*-frame to the *p*-frame is:

(4) $$C_{b}^{p}=\left[\begin{array}{ccc} -\cos S_{t}\sin h\cos q-\sin S_{t}\sin q & \cos S_{t}\sin h\sin q-\sin S_{t}\cos q & -\cos S_{t}\cos h\\ -\sin S_{t}\sin h\cos q+\cos S_{t}\sin q & \sin S_{t}\sin h\sin q+\cos S_{t}\cos q & -\sin S_{t}\cos h\\ \cos h\cos q & -\cos h\sin q & -\sin h \end{array}\right].$$

In Equation (3), ψ, θ, and ϕ are yaw, pitch, and roll, respectively. In Equation (4), $S_{t}$ is the rotation angle of the platform’s revolution axis relative to the revolution axis of the inner gimbal, *h* is the rotation angle of the revolution axis of the middle gimbal relative to that of the outer gimbal, and *q* is the rotation angle of the revolution axis of the outer gimbal relative to the base. The rotation angle of the revolution axis of the inner gimbal relative to that of the middle gimbal is zero, for the outer gimbal is tracking the rotation angle of the inner gimbal, and for the revolution axes of the platform, the inner gimbal and the middle gimbal are orthogonal.

**Figure 1:**
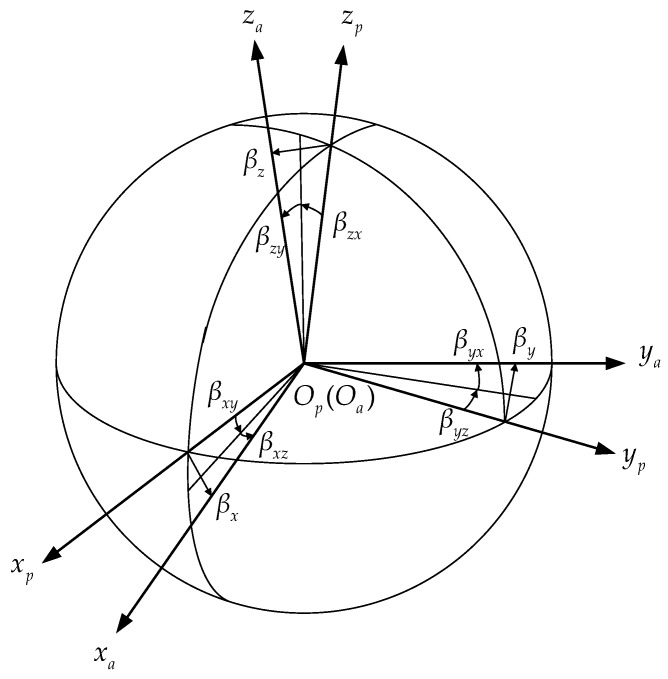
The geometric relationship between the *p*-frame and the *a*-frame.

The geometric relationship between the orthogonal *p*-frame and the non-orthogonal *a*-frame is shown in Figure 1. It is assumed that installation error angles are all small quantities. $\beta_{x},\;\beta_{y}\;\mathrm{and}\;\beta_{z}$ denote the three installation error angles in the *x*-direction, *y*-direction, and *z*-direction, respectively. Each of these three small angles can be decomposed into two rotation angles around the axes of the *p*-frame, namely $\beta_{xy}\;\mathrm{and}\;\beta_{xz}$, $\beta_{yz}\;\mathrm{and}\;\beta_{yx},$ and $\beta_{zx}\;\mathrm{and}\;\beta_{zy}$.

According to the geometric relationship shown in Figure 1, the direction cosine matrix from the *p*-frame to the *a*-frame can be calculated as:

(5) $$C_{p}^{a}=\left[\begin{array}{ccc} 1 & \beta_{xz} & -\beta_{xy}\\ -\beta_{yz} & 1 & \beta_{yx}\\ \beta_{zy} & -\beta_{zx} & 1 \end{array}\right].$$

Thus the specific force measured by the tri-axis accelerometer satisfies an equation, as:

(6) $$f^{a}=SF^{-1}C_{p}^{a}C_{b}^{p}C_{n}^{b}f^{n}+\delta b+K_{nl}{\left(f^{a}-\delta b\right)}^{2},$$

where ***SF*** is the matrix of scale factors, δb is the bias vector, and $K_{nl}$ is the matrix of nonlinear coefficients of the scale factors. In this paper, a three-stage calibration technique is proposed, to estimate and compensate all the parameters shown in Equation (6). The first two stages will lay a foundation of full-parameter and full-value estimations of model coefficients. The third stage will then correct part of the coefficients and further improve the calibration accuracy. Although the final calibration accuracy will mainly depend on the novel system-level calibration method, it must work on the basis of the device-level calibration. Therefore, the first two stages of the device-level calibration designed for the space-stable INS will also be introduced to form the integrated three-stage calibration technique.

## 3. Device-Level Coarse Calibration

**Figure 2:**
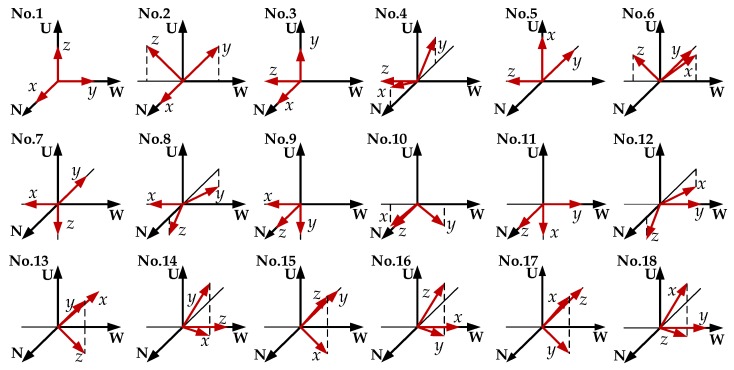
The 18 positions chosen for accelerometer calibration.

For the tri-axis accelerometer, there are totally 18 error coefficients that need to be calibrated: three scale factors, ${SF}_{x},\;{SF}_{y}\;\mathrm{and}\;{SF}_{z}$; three biases, ${\delta b}_{x},\;{\delta b}_{y}\;\mathrm{and}\;{\delta b}_{z}$; three nonlinear coefficients of the scale factors, $K_{nlx},\;K_{nly}\;\mathrm{and}\;K_{nlz}$; six installation error angles $\beta_{xy},\;\beta_{xz}$, $\beta_{yz},\;\beta_{yx},\;\beta_{zx}\;\mathrm{and}\;\beta_{zy}$; and three zero-point offsets of the gimbals’ angular sensors, $\theta_{10}$ of the platform axis, $\theta_{20}$ of the inner gimbal axis, and $\theta_{30}$ of the middle gimbal axis. The platform needs to be rotated to 18 positions. Table 1 lists the order of rotation, and the corresponding rotation angles of the gimbals and components of the *p*-frame gravity vector [3], where the symbol ↓ denotes that the angle remains unchanged. Figure 2 shows the directions of the accelerometer’s sensitive axes in detail, where N, W, and U denote the local northern, western and up directions, respectively, and *x*, *y*, and *z* denote the orientations of the tri-axis accelerometer.

During coarse calibration, the nonlinearity of scale factors is ignored. Thus the accelerometer measurement equation shown in Equation (6) can be simplified as:

(7) $$f^{a}=SF^{-1}C_{p}^{a}g^{p}+\delta b,$$

where $C_{p}^{a}g^{p}$ is the gravity vector sensed by the tri-axis accelerometer.

Each position takes 20 s for the rapid measurement. Based on the outputs of the tri-axis accelerometer at different positions and Table 1, 18 equations can be obtained according to Equation (7) to preliminarily estimate 15 parameters except the nonlinear coefficients. Scale factors and biases can be coarsely estimated by solving the equation set corresponding to the six rotation positions (No. 1, 3, 5, 7, 9, and 11) where each sensitive axis of the tri-axis accelerometer points upward or downward along the $z_{n}$ axis in turn. The other equations corresponding to the rest of the rotation positions (No. 2, 4, 6, 8, 10, and 12~18), where one of the sensitive axes is parallel to the $x_{n}$ axis or the $y_{n}$ axis, and other two sensitive axes have included angles of 45° with the vertical direction, are used to estimate zero-point offsets of the angular sensors and installation error angles.

Estimated values of zero-point offsets, ${\hat{\theta }}_{20}$ and ${\hat{\theta }}_{30}$, are used to coarsely correct the orientation of the *p*-frame, and to make the directions of the middle gimbal axis and the inner gimbal axis approach the directions of axes of the *p*-frame. Concretely, the inner gimbal axis and the middle gimbal axis are inversely rotated ${\hat{\theta }}_{20}$ and ${\hat{\theta }}_{30}$, respectively, and then coarse calibration is re-executed. This procedure will repeat until the re-estimated ${\hat{\theta }}_{20}$ and ${\hat{\theta }}_{30}$ decrease to negligibly small quantities.

## 4. Device-Level Fine Calibration

Fine calibration will be executed on the basis of coarse calibration. The orientation of the platform has been corrected and new data will be measured in more accurate rotation positions. In order to improve the data collection accuracy, the measurement time of each position is extended to 10 min. Moreover, to improve the data processing accuracy, the estimation error of coarse calibration is estimated instead of the full values of error parameters.

According to Equation (6), the ideal specific force in the *p*-frame satisfies:

(8) $$f^{p}=C_{a}^{p}SF\left(f^{a}-\delta b-K_{nl}{\left(f^{a}-\delta b\right)}^{2}\right).$$

If the result of coarse calibration and outputs of the tri-axis accelerometer during fine calibration ($f^{a}$) have been obtained, the coarse estimation of $f^{p}$ can be calculated as:

(9) $${\hat{f}}^{p}={\hat{C}}_{a}^{p}\hat{SF}\left(f^{a}-\hat{\delta b}\right),$$

where “hatted” symbols denote the estimated values.

In order to reduce the requirement for position control accuracy of the platform during fine calibration, the observed variable is not the direct difference between the coarsely estimated value and the ideal value of the specific force at one certain position, but the difference between the squared amplitudes of them. Subtracting the squared amplitude of Equation (9) from that of Equation (8), and then taking half of the result yields the observed variable, as:

(10) $$\begin{array}{cc} \frac{1}{2}\left({\left(f^{p}\right)}^{T}f^{p}-{\left({\hat{f}}^{p}\right)}^{T}{\hat{f}}^{p}\right) & ={\left(\hat{SF}\left(f^{a}-\delta b\right)\right)}^{2}{\hat{SF}}^{-1}\Delta SF\\ & +\frac{1}{2}{\left(\hat{SF}\left(f^{a}-\delta b\right)\right)}^{T}\left({\left(\Delta C_{p}^{a}\right)}^{T}+\Delta C_{p}^{a}\right)\left(\hat{SF}\left(f^{a}-\delta b\right)\right)\\ & -{\left(\hat{SF}\left(f^{a}-\delta b\right)\right)}^{T}\hat{SF}\tilde{\delta b}-{\left(\hat{SF}\left(f^{a}-\delta b\right)\right)}^{T}\hat{SF}K_{\mathrm{nl}}\left(f^{a}-\delta b\right)\left(f^{a}-\delta b\right) \end{array},$$

where:

(11) $$\frac{1}{2}\left({\left(f^{p}\right)}^{T}f^{p}-{\left({\hat{f}}^{p}\right)}^{T}{\hat{f}}^{p}\right)=\frac{1}{2}\left({\left(f^{p}\right)}^{2}-{\left({\hat{f}}^{p}\right)}^{2}\right)\approx f^{p}\left(f^{p}-{\hat{f}}^{p}\right),$$

(12) $$\Delta SF=SF-\hat{SF}=\left[\begin{array}{ccc} \Delta SF_{x} & 0 & 0\\ 0 & \Delta SF_{y} & 0\\ 0 & 0 & \Delta SF_{z} \end{array}\right],$$

(13) $$\tilde{\delta b}=\delta b-\hat{\delta b}=\left[\begin{array}{ccc} \tilde{\delta b_{x}} & 0 & 0\\ 0 & \tilde{\delta b_{y}} & 0\\ 0 & 0 & \tilde{\delta b_{z}} \end{array}\right],$$

(14) $$\Delta C_{p}^{a}=\left[\begin{array}{ccc} 0 & -\Delta \beta_{xz} & \Delta \beta_{xy}\\ \Delta \beta_{yz} & 0 & -\Delta \beta_{yx}\\ -\Delta \beta_{zy} & \Delta \beta_{zx} & 0 \end{array}\right],\;\mathrm{and}$$

(15) $${\hat{f}}^{a}=\hat{SF}\left(f^{a}-\delta b\right)=\left(\begin{array}{c} {\hat{f}}_{x}^{a}\\ {\hat{f}}_{y}^{a}\\ {\hat{f}}_{z}^{a} \end{array}\right).$$

According to Equations (10)–(15), the observation equation at position *j* can be written as:

(16) $$\begin{array}{c} {}[\begin{array}{ccccccccc} {\left({\hat{f}}_{xj}^{a}\right)}^{2} & {\left({\hat{f}}_{yj}^{a}\right)}^{2} & {\left({\hat{f}}_{zj}^{a}\right)}^{2} & -{\hat{f}}_{xj}^{a} & -{\hat{f}}_{yj}^{a} & -{\hat{f}}_{zj}^{a} & -{\left({\hat{f}}_{xj}^{a}\right)}^{3} & -{\left({\hat{f}}_{yj}^{a}\right)}^{3} & -{\left({\hat{f}}_{zj}^{a}\right)}^{3} \end{array}\\ \begin{array}{ccc} \;{\hat{f}}_{xj}^{a}{\hat{f}}_{yj}^{a} & {\hat{f}}_{xj}^{a}{\hat{f}}_{zj}^{a} & {\hat{f}}_{zj}^{a}{\hat{f}}_{yj}^{a} \end{array}]dx=f^{p}\left(f^{p}-{\hat{f}}_{j}^{p}\right) \end{array},$$

where the unknown error vector is:

(17) $$\begin{array}{cc} dx= & [\begin{array}{cccccc} \frac{\hat{\Delta SF_{x}}}{\hat{SF_{x}}} & \frac{\hat{\Delta SF_{y}}}{\hat{SF_{y}}} & \frac{\hat{\Delta SF_{z}}}{\hat{SF_{z}}} & \hat{SF_{x}}{\tilde{\Delta b}}_{x} & \hat{SF_{y}}{\tilde{\Delta b}}_{y} & \hat{SF_{z}}{\tilde{\Delta b}}_{z} \end{array}\\ & \begin{array}{cccccc} \frac{K_{\mathrm{nlx}}}{\hat{SF_{x}}} & \frac{K_{\mathrm{nly}}}{\hat{SF_{y}}} & \frac{K_{\mathrm{nlz}}}{\hat{SF_{z}}} & \Delta \beta_{yz}-\Delta \beta_{xz} & \Delta \beta_{xy}-\Delta \beta_{zy} & \Delta \beta_{zx}-\Delta \beta_{yx} \end{array}{]}^{T} \end{array}.$$

Based on the data collected at the 18 positions, an 18×12-dimension observation equation can be formed based on Equation (16), and the unknown variables shown in Equation (17) can be solved by the least squares method. Finally, using scale factors to convert the solved dx and adding the result to the coarse estimation will yield a full-value fine estimation of 12 error coefficients. It should be noted that $\beta_{xz}\;\mathrm{and}\;\beta_{yz}$, $\beta_{zy}\;\mathrm{and}\;\beta_{xy},\;\mathrm{and}\;\beta_{yx}\;\mathrm{and}\;\beta_{zx}$ are linearly correlated in pairs and cannot be decomposed from each other. Therefore the fine calibration can only improve the accuracy of $f^{p}$, but it cannot calibrate the rotation characteristics of the *a*-frame.

## 5. System-Level Calibration Based on Specific Force Error and Attitude Error

Errors of inertial instruments will induce the navigational errors of INSs, including the positional error, the velocity error and the attitude error. Inversely, the independently observed navigation errors such as the specific force error, the attitude error and the position error can be used to calibrate the main error parameters in the system. As the position error is affected by both the gyroscope error and accelerometer error, it is generally difficult to directly extract the accelerometer-induced term from the position error. On the other hand, the specific force error and the horizontal attitude error are mainly caused by the accelerometer error. Therefore this paper employs the specific force and horizontal attitude angles to correct part of the accelerometer’s error coefficients. The prerequisite for accurate observation in such a system-level calibration is that the system should be installed on a static and horizontal base.

### 5.1. Calibration Using Specific Force Error

Under the ideal condition, that there is no installation error between the base and the stabilized platform, and that the measurement of gimbals’ rotation angle is accurate, there is an approximate relationship:

(18) $$C_{b}^{p}C_{n}^{b}\approx \left[\begin{array}{ccc} -\cos S_{t}\sin L & -\sin S_{t} & -\cos S_{t}\cos L\\ -\sin S_{t}\sin L & \cos S_{t} & -\sin S_{t}\cos L\\ \cos L & 0 & -\sin L \end{array}\right],$$

where *L* denotes the latitude. On the static and horizontal base, the local scalar gravity *g* can be accurately calculated. Then, according to Equations (1) and (18), the ideal specific force vector in the *p*-frame can be written as:

(19) $$\frac{f^{p}}{g}=\left(\begin{array}{c} f_{x}^{P}/g\\ f_{y}^{P}/g\\ f_{z}^{P}/g \end{array}\right)=C_{b}^{p}C_{n}^{b}\left(\begin{array}{c} 0\\ 0\\ -1 \end{array}\right)=\left(\begin{array}{c} \cos S_{t}\cos L\\ \sin S_{t}\cos L\\ \sin L \end{array}\right).$$

Similar to the device-level calibration method presented in the previous section, the difference of the squared amplitudes of the measured and the ideal specific force vectors is chosen as the observed variable, as:

(20) $$z=\frac{1}{2}\left[{\left|\frac{{\hat{f}}^{p}}{g}\right|}^{2}-{\left|\frac{f^{p}}{g}\right|}^{2}\right]\approx {\left(\frac{f^{p}}{g}\right)}^{T}\frac{\Delta f^{p}}{g}.$$

A 5-dimensional state vector is constructed as $x={\left[\begin{array}{ccccc} x_{1} & x_{2} & x_{3} & x_{4} & x_{5} \end{array}\right]}^{T}$, where:

(21) $$x_{1}=\left(\frac{\Delta SF_{x}}{2SF_{x}}+\frac{\Delta SF_{y}}{2SF_{y}}\right)\cos^{2}L-\left(\frac{{\tilde{\delta b}}_{z}}{g}-\frac{\Delta SF_{z}}{SF_{z}}\sin L\right)\sin L,$$

(22) $$x_{2}=\frac{{\tilde{\delta b}}_{x}}{g}-\Delta \beta_{xy}\sin L+\Delta \beta_{zy}\sin L,$$

(23) $$x_{3}=\frac{{\tilde{\delta b}}_{y}}{g}+\Delta \beta_{yx}\sin L-\Delta \beta_{zx}\sin L,$$

(24) $$x_{4}=\frac{\Delta SF_{x}}{2SF_{x}}-\frac{\Delta SF_{y}}{2SF_{y}},\;\mathrm{and}$$

(25) $$x_{5}=\frac{\Delta \beta_{yz}-\Delta \beta_{xz}}{2}.$$

Thus the observation equation is:

(26) z=Hx+v.

Considering Equation (19), the observation matrix is:

(27) $$H=\left[\begin{array}{ccccc} 1 & -f_{x}^{p}/g & -f_{y}^{p}/g & \left({\left(f_{x}^{p}\right)}^{2}-{\left(f_{y}^{p}\right)}^{2}\right)/g^{2} & 2f_{x}^{p}f_{y}^{p}/g^{2} \end{array}\right].$$

The five components of x are actually the mean value, the sine and the cosine fundamental waves, and the sine and the cosine second harmonics of the Earth’s rotation rate in the specific force error. Assuming that the observation noise is a zero-mean Gaussian white noise, x can be estimated by a Kalman filter.

### 5.2. Calibration Using Attitude Error

On the static and horizontal base, the attitude error of the INS with both vertical damping and horizontal velocity damping mainly consists of a constant value and a 24 h long period component, which is induced by the gyroscope error, the accelerometer error, and the rotation angle errors of platform’s gimbals. Using the space-stable mechanization, the azimuth error will accumulate over time due to the gyroscope error, while the horizontal attitude errors are mainly caused by the accelerometer error and have no such accumulation. Therefore, the horizontal attitude errors are chosen as the observed quantities for accelerometer calibration.

Neglecting the short-period misalignment angle of gyro case rotation, the attitude error on the static and horizontal base has an expression as:

(28) $$\left(\begin{array}{c} \delta \phi \\ \delta \theta \\ \delta \psi \end{array}\right)=\left(\begin{array}{c} \frac{\Delta f_{y}^{e}}{g}\\ \frac{\Delta f_{x}^{e}\sin L-\Delta f_{z}^{e}\cos L}{g}\\ -\frac{\Delta S_{1}}{\cos L}-\frac{\Delta f_{y}^{e}}{g}\tan L \end{array}\right)-\left(\begin{array}{c} \delta S_{t}\cos L+\delta \kappa_{x}^{b}\cos\psi -\delta \kappa_{y}^{b}\sin\psi \\ -\delta h+\delta \kappa_{x}^{b}\sin\psi +\delta \kappa_{y}^{b}\cos\psi \\ \delta q-\delta S_{t}\sin L+\delta \kappa_{z}^{b} \end{array}\right),$$

where ${\delta \kappa }_{x}^{b}$, ${\delta \kappa }_{y}^{b}$, and ${\delta \kappa }_{z}^{b}$ are the transverse tilt error, the longitudinal tilt error, and the azimuth error of the base, respectively, and the superscript *e* denotes the earth-fixed frame (*e*-frame).

Substituting the *e*-frame specific force error into Equation (28) yields the expressions of pitch error and roll error. Both of them consist of the mean value, the fundamental wave, and the second harmonic of the Earth’s rotation rate. The five Fourier coefficients of the pitch error are:

(29) $$\bar{\delta \theta }=-\left(-\delta h+\delta \kappa_{x}^{b}\sin\psi +\delta \kappa_{y}^{b}\cos\psi \right)+\left[\frac{\nabla_{z}}{g}-\left(\frac{\Delta SF_{z}}{SF_{z}}-\frac{\Delta SF_{x}}{2SF_{x}}-\frac{\Delta SF_{y}}{2SF_{y}}\right)\sin L\right]\cos L,$$

(30) $$\tilde{\delta \theta_{1c}}=-\left(\frac{\nabla_{x}}{g}\sin L-\Delta \beta_{xy}\sin^{2}L-\Delta \beta_{zy}\cos^{2}L\right),$$

(31) $$\tilde{\delta \theta_{1s}}=-\left(\frac{\nabla_{y}}{g}\sin L+\Delta \beta_{yx}\sin^{2}L+\Delta \beta_{zx}\cos^{2}L\right),$$

(32) $$\tilde{\delta \theta_{2c}}=\left(\frac{\Delta SF_{x}}{2SF_{x}}-\frac{\Delta SF_{y}}{2SF_{y}}\right)\sin L\cos L,\;\mathrm{and}$$

(33) $$\tilde{\delta \theta_{2s}}=\left(\frac{\Delta \beta_{yz}}{2}-\frac{\Delta \beta_{xz}}{2}\right)\sin L\cos L.$$

The other five Fourier coefficients of the roll error are:

(34) $$\bar{\delta \phi }=-\left(\delta S_{t}\cos L+\delta \kappa_{x}^{b}\cos\psi -\delta \kappa_{y}^{b}\sin\psi \right)+\left(\frac{\Delta \beta_{yz}}{2}+\frac{\Delta \beta_{xz}}{2}\right)\cos L,$$

(35) $$\tilde{\delta \phi_{1s}}=\frac{\nabla_{x}}{g}-\Delta \beta_{xy}\sin L,$$

(36) $$\tilde{\delta \phi_{1c}}=-\frac{\nabla_{y}}{g}-\Delta \beta_{yx}\sin L,$$

(37) $$\tilde{\delta \phi_{2s}}=-\left(\frac{\Delta SF_{x}}{2SF_{x}}-\frac{\Delta SF_{y}}{2SF_{y}}\right)\cos L,\;\mathrm{and}$$

(38) $$\tilde{\delta \phi_{2c}}=\left(\frac{\Delta \beta_{yz}}{2}-\frac{\Delta \beta_{xz}}{2}\right)\cos L.$$

The subscripts indicate which component each expression is (1 for the fundamental waves, 2 for the second harmonics, s for the sine functions, and c for the cosine functions). After the error curves of pitch and roll are obtained, these coefficients can be extracted by Fourier expansion.

### 5.3. Parameter Separation Algorithm for Combined Error Coefficients of Accerelometer

In Section 5.1 and Section 5.2, 15 combined coefficients (Equations (21)–(25), (29)–(33), and (34)–(38)) are estimated by static Kalman filter and Fourier expansion. Twelve of them are selected and divided into three groups to extract the residual error parameters of the tri-axis accelerometer. Since only the fundamental waves and second harmonics of pitch and roll errors are concerned, the requirement for the horizontality of the base need not be very strict.

The first group consists of three equations, which are the coefficients of the sine and cosine second harmonics of Earth’s rotation rate about $\frac{{\Delta SF}_{x}}{{2SF}_{x}}-\frac{{\Delta SF}_{y}}{{2SF}_{y}}$. They are the linearly correlated Equations (24), (32), and (37). Thus $\frac{{\Delta SF}_{x}}{{2SF}_{x}}-\frac{{\Delta SF}_{y}}{{2SF}_{y}}$ can be solved using their equally weighted average, as:

(39) $$\hat{\frac{\Delta SF_{x}}{2SF_{x}}}-\hat{\frac{\Delta SF_{y}}{2SF_{y}}}=\frac{1}{3}\left(x_{4}-\frac{\phi_{N}{}_{2s}}{\cos L}+\frac{\phi_{E}{}_{2c}}{\cos L\sin L}\right).$$

Similarly, the second group consists of Equations (25), (33), and (38), and $\frac{{\Delta \beta }_{yz}{-\Delta \beta }_{xz}}{2}$ can be solved as:

(40) $$\frac{\hat{\Delta \beta_{yz}}-\hat{\Delta \beta_{xz}}}{2}=\frac{1}{3}(x_{5}+\frac{\phi_{N}{}_{2c}}{\cos L}+\frac{\phi_{E}{}_{2s}}{\cos L\sin L}).$$

Another six parameters ($\frac{{𝛻}_{x}}{g}$, $\frac{{𝛻}_{y}}{g}$, ${\Delta \beta }_{yx}$, ${\Delta \beta }_{zx}$, ${\Delta \beta }_{xy}$, ${\Delta \beta }_{zy}$) can be solved using the least square method based on the remaining six combined coefficients, according to:

(41) $$\left[\begin{array}{cccccc} 1 & 0 & 0 & 0 & -\sin L & 0\\ 0 & -1 & -\sin L & 0 & 0 & 0\\ -\sin L & 0 & 0 & 0 & \sin^{2}L & \cos^{2}L\\ 0 & -\sin L & -\sin^{2}L & -\cos^{2}L & 0 & 0\\ 1 & 0 & 0 & 0 & 0 & 0\\ 0 & 1 & 0 & 0 & 0 & 0 \end{array}\right]\left[\begin{array}{c} {𝛻}_{x}/g\\ {𝛻}_{y}/g\\ \Delta \beta_{yx}\\ \Delta \beta_{zx}\\ \Delta \beta_{xy}\\ \Delta \beta_{zy} \end{array}\right]=\left[\begin{array}{c} \tilde{\delta \phi_{1s}}\\ \tilde{\delta \phi_{1c}}\\ \tilde{\delta \theta_{1c}}\\ \tilde{\delta \theta_{1s}}\\ \hat{x_{2}}\\ \hat{x_{3}} \end{array}\right].$$

The six parameters in Equation (41) can be directly compensated by substituting them into the accelerometer’s error model. Also, $\frac{{\Delta \beta }_{yz}{-\Delta \beta }_{xz}}{2}$ and $\frac{{\Delta SF}_{x}}{{2SF}_{x}}-\frac{{\Delta SF}_{y}}{{2SF}_{y}}$ will be indirectly compensated by deducting the error terms about them from the *n*-frame specific force expression.

## 6. Simulation and Experiment

### 6.1. Simulation Result

As the final accuracy of the three-stage calibration mainly depends on the performance of the last stage, the simulation of the system-level calibration is conducted first. In the simulation, the location of the static base is set at 40° N. The non-orthogonal installation error of the tri-axis accelerometer is quite a stable mechanical error, and after the 18-position two-stage device-level calibration, the residuals are generally less than 10″. Thus, the residuals of accelerometer’s error parameters are assigned as Table 2 and Table 3. In addition, a zero-mean random noise with an amplitude of ${5\times 10}^{-3}\;{m/s}^{2}$ is added to each sensitive axis of the tri-axis accelerometer.

Truth values of the system-level combined coefficients are calculated according to the error parameters shown in Table 2 and Table 3, and are listed in Table 4 as reference values. The combined coefficients are estimated by system-level calibration based on specific force error and attitude error, and are also listed in Table 4. Comparison shows that the estimation accuracy of each combined coefficient is better than $10^{-6}\;\mathrm{rad}$, which is equivalent to an accelerometer calibration accuracy that is better than $10^{-6}$ g. It indicates that the method can be used for the accelerometer calibration of high-precision INSs.

### 6.2. Experiment Results

In order to validate the proposed three-stage accelerometer calibration method, a static-base navigation test has been conducted using a high-precision space-stable INS. The INS is in the navigation grade class and has been introduced in reference [36]. Two dual-axis free-rotor gyros and a tri-axis quartz accelerometer are mounted on a gimbaled platform. According to the outputs of the free-rotor gyros, the platform is servo-controlled to be stabilized in the inertial space. The gyros have an ultra-low drift of less than 0.001°/h. The measurement error of the accelerometer is of a ${1\times 10}^{-5}$ g order of magnitude, and its stability is better than ${1\times 10}^{-5}$ g per year. The static measurement accuracy of gimbals’ rotation angles can reach the level of 1″. The position error of this navigation system can reach the order of magnitude of 1 n mile per day. The data used in the experiment are collected when the system is installed on a static and horizontal base.

**Figure 3:**
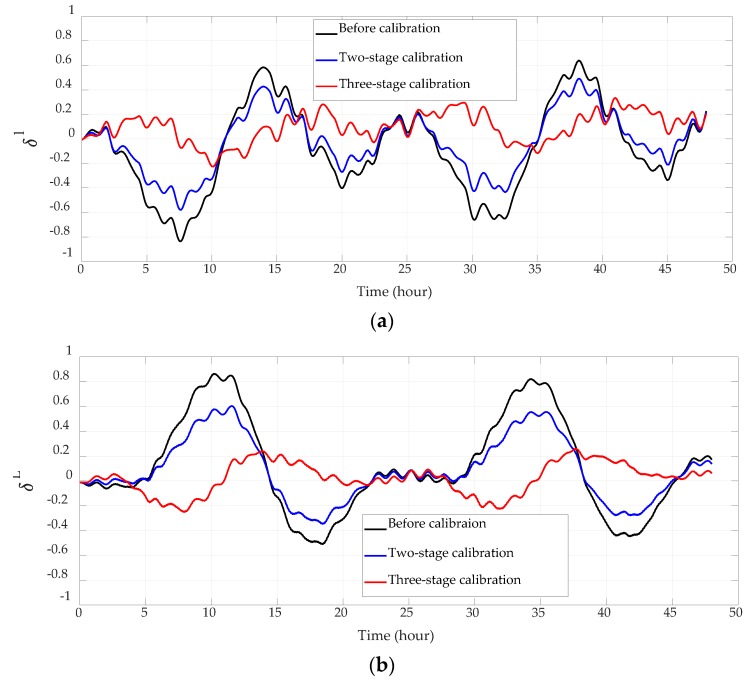
Horizontal position errors. (**a**) Longitude errors; (**b**) latitude errors.

**Figure 4:**
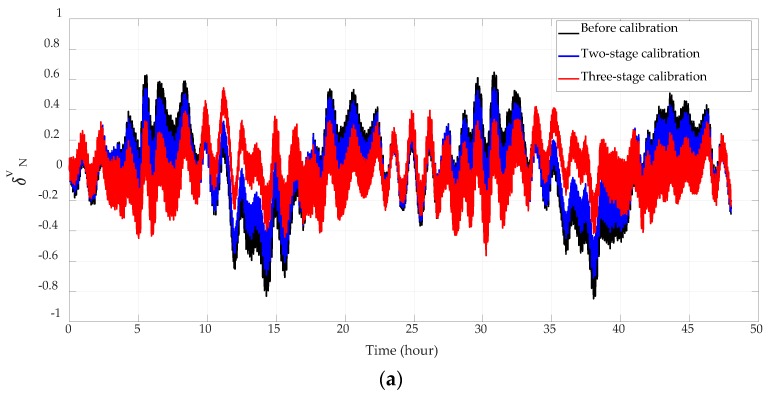

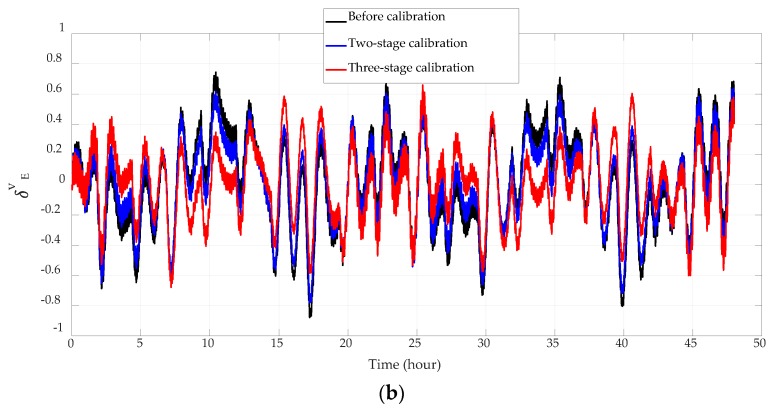
Horizontal velocity errors. (**a**) Northern velocity errors; (**b**) eastern velocity errors.

**Figure 5:**
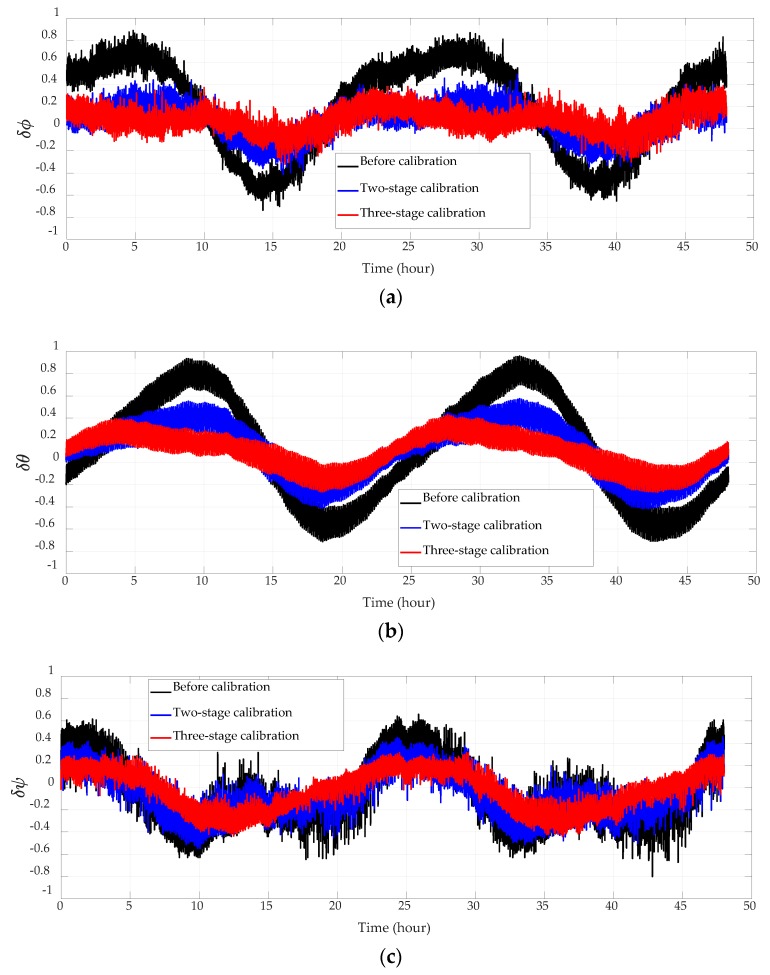
Attitude errors. (**a**) Roll errors; (**b**) pitch errors; (**c**) yaw errors.

Navigation errors before calibration, after the original two-stage 18-position coarse and fine self-calibration, and after the entire three-stage calibration are compared in Figure 3, Figure 4 and Figure 5 and Table 5. Since the system employs vertical damping, height error and vertical velocity error can be neglected and are not presented. The output position of the system is compared with the reference position of the base to obtain the position error. The output velocity and horizontal attitude angles are actually the velocity error and horizontal attitude error, for the base is static and horizontal. All experiment results here have been normalized.

The system has employed gyro case rotation techniques to decrease the gyro drift. Although its influence on the platform’s motion has been deducted during navigation calculating, there are still 8-minute residuals in the velocity and attitude error curves. As the cycle of these residuals are much shorter than the test time, they are severely squeezed in the error curves shown in Figure 4 and Figure 5.

Accelerometer parameters will change after the high-precision INS works for a period of time, which will consequently lead to the increase of navigation errors. Therefore, on-site and online calibration is necessary to improve the navigation precision. Latitude error, longitude error, and attitude errors are reduced by over 30% after the two-stage calibration, and by over 45% after the three-stage calibration. Since the velocity error mainly consists of Schuler oscillation, and the error term is related to the gyro case rotation residual, the improvement of velocity accuracy is not conspicuous. The velocity error has decreased by over 10% after the two-stage calibration, and by over 22% after the three-stage calibration.

## 7. Conclusions

A three-stage tri-axis accelerometer self-calibration technique is proposed with a thorough theoretical and mathematical analysis. Compared with existing calibration methods, the proposed three-stage calibration method has the following features and advantages: (1) Taking full advantage of high-precision angulation of the gimbaled platform in the space-stable INS, the two-stage device-level calibration is implemented directly on the platform of the space-stable INS. Thus the entire three-stage self-calibration can be implemented in the INS after the tri-axis accelerometer has been mounted on the platform. Besides, during the device-level calibration, zero-point offsets of the gimbals’ angular sensors are simultaneously estimated and used to correct the platform’s orientation. This will help improve the system’s navigation performance. (2) Different from the existing calibration methods which usually estimate error parameters of both accelerometer and gyroscope simultaneously, the proposed system-level calibration is designed based on error propagation to choose observed quantities only resulting from accelerometer measurement error. This can get rid of the influence of gyro drifts. (3) Combining device-level calibration and system-level calibration, the proposed method can achieve both full-parameter estimation of the accelerometer measurement model and high-precision correction for part of the parameters, while existing calibration methods normally concentrate on one of the two aspects. Both simulation and experiment results validate the proposed method. The simulation result shows that using the proposed three-stage calibration technique, the estimation accuracy of accelerometer error is better than ${1\times 10}^{-6}$ g. Experiment results show that after implementing the three-stage calibration, during navigation, the accuracy of latitude, longitude, and attitude angles has increased by over 45%, and the accuracy of velocity has increased by over 22%.

## Figures and Tables

**Table 1:** The order of rotation for the 18-position calibration, and the corresponding rotation angles of the gimbals and components of the *p*-frame gravity vector.

The Order of Rotation	Rotation Angles of Gimbals			Components of the *p*-Frame Gravity Vector		
	$\theta_{1}$	$\theta_{2}$	$\theta_{3}$	$g_{x}^{p}$	$g_{y}^{p}$	$g_{z}^{p}$
1	$\theta_{10}$	$\theta_{20}$	$\theta_{30}$	${-g\theta }_{30}$	${g\theta }_{20}$	g
2	↓	$\theta_{20}+\frac{\pi }{4}$	↓	$(\frac{\sqrt{2}}{2}\theta_{10}{-\theta }_{30})g$	$\frac{\sqrt{2}}{2}g$	$\frac{\sqrt{2}}{2}g$
3	↓	$\theta_{20}+\frac{\pi }{2}$	↓	${g(\theta }_{10}{-\theta }_{30})$	g	${-g\theta }_{20}$
4	$\theta_{10}+\frac{\pi }{4}$	↓	↓	$\frac{\sqrt{2}}{2}g$	$\frac{\sqrt{2}}{2}g$	${-g\theta }_{20}$
5	$\theta_{10}+\frac{\pi }{2}$	↓	↓	g	${g(\theta }_{30}{-\theta }_{10})$	${-g\theta }_{20}$
6	↓	$\theta_{20}+\frac{\pi }{4}$	↓	$\frac{\sqrt{2}}{2}g$	${(\theta }_{30}-\frac{\sqrt{2}}{2}\theta_{10})g$	$\frac{\sqrt{2}}{2}g$
7	↓	$\theta_{20}+\pi$	↓	${-g\theta }_{20}$	${g\theta }_{30}$	−g
8	↓	↓	$\theta_{30}-\frac{\pi }{4}$	$-\frac{\sqrt{2}}{2}{g(\theta }_{10}{+\theta }_{20})$	$-\frac{\sqrt{2}}{2}g$	$-\frac{\sqrt{2}}{2}g$
9	↓	↓	$\theta_{30}-\frac{\pi }{2}$	${-g\theta }_{10}$	−g	${-g\theta }_{30}$
10	$\theta_{10}+\frac{3\pi }{4}$	↓	↓	$-\frac{\sqrt{2}}{2}g$	$-\frac{\sqrt{2}}{2}g$	${-g\theta }_{30}$
11	$\theta_{10}+\pi$	↓	↓	−g	${g\theta }_{10}$	${-g\theta }_{30}$
12	↓	↓	$\theta_{30}-\frac{\pi }{4}$	$-\frac{\sqrt{2}}{2}g$	$\frac{\sqrt{2}}{2}{g(\theta }_{10}{+\theta }_{20})$	$-\frac{\sqrt{2}}{2}g$
13	↓	$\theta_{20}+\frac{5\pi }{4}$	$\theta_{30}$	$(\frac{\sqrt{2}}{2}\theta_{10}{+\theta }_{30})g$	$\frac{\sqrt{2}}{2}g$	$-\frac{\sqrt{2}}{2}g$
14	$\theta_{10}+\frac{3\pi }{4}$	$\theta_{20}+\frac{3\pi }{2}$	↓	$-\frac{\sqrt{2}}{2}g$	$\frac{\sqrt{2}}{2}g$	$\frac{\sqrt{2}}{2}{g\theta }_{20}$
15	$\theta_{10}+\frac{\pi }{2}$	$\theta_{20}+\frac{7\pi }{4}$	↓	$-\frac{\sqrt{2}}{2}g$	$\frac{\sqrt{2}}{2}{g(\theta }_{30}{+\theta }_{20})$	$\frac{\sqrt{2}}{2}g$
16	↓	$\theta_{20}$	$\theta_{30}-\frac{\pi }{4}$	$\frac{\sqrt{2}}{2}{g(-\theta }_{10}{+\theta }_{20})$	$-\frac{\sqrt{2}}{2}g$	$\frac{\sqrt{2}}{2}g$
17	$\theta_{10}+\frac{\pi }{4}$	↓	$\theta_{30}-\frac{\pi }{2}$	$\frac{\sqrt{2}}{2}g$	$-\frac{\sqrt{2}}{2}g$	${g\theta }_{30}$
18	$\theta_{10}$	↓	$\theta_{30}-\frac{3\pi }{4}$	$\frac{\sqrt{2}}{2}g$	$-\frac{\sqrt{2}}{2}{g(\theta }_{10}{+\theta }_{20})$	$-\frac{\sqrt{2}}{2}g$

**Table 2:** Residuals of scale factor errors and biases.

$\Delta {\mathrm{SF}}_{x}/{\mathrm{SF}}_{x}$	$\Delta {\mathrm{SF}}_{y}/{\mathrm{SF}}_{y}$	$\Delta {\mathrm{SF}}_{z}/{\mathrm{SF}}_{z}$	$\nabla_{x}$	$\nabla_{y}$	$\nabla_{z}$
$10^{-4}$	${2\times 10}^{-4}$	${-10}^{-4}$	${-10}^{-3}\;{m/s}^{2}$	${5\times 10}^{-4}\;{m/s}^{2}$	$10^{-3}\;{m/s}^{2}$

**Table 3:** Residuals of installation error angles.

${\Delta \beta }_{\mathrm{xz}}$	${\Delta \beta }_{\mathrm{yz}}$	${\Delta \beta }_{\mathrm{zy}}$	${\Delta \beta }_{\mathrm{xy}}$	${\Delta \beta }_{\mathrm{yx}}$	${\Delta \beta }_{\mathrm{zx}}$
5″	10″	−5″	−10″	5″	−5″

**Table 4:** Simulation result of system-level calibration of the combined accelerometer error coefficients (unit: rad).

Combined Coefficients	Reference Values	Estimated Values	Errors
$x_{1}$	$1.1228\times 10^{-4}$	$1.1256\times 10^{-4}$	$2.8\times 10^{-7}$
$x_{2}$	$-8.645\times 10^{-5}$	$-8.612\times 10^{-5}$	$3.3\times 10^{-7}$
$x_{3}$	$8.217\times 10^{-5}$	$8.206\times 10^{-5}$	$-1.1\times 10^{-7}$
$x_{4}$	$-5.000\times 10^{-5}$	$-4.957\times 10^{-5}$	$4.3\times 10^{-7}$
$x_{5}$	$1.212\times 10^{-5}$	$1.185\times 10^{-5}$	$-2.7\times 10^{-7}$
$\delta \phi_{1s}$	$-1.3319\times 10^{-4}$	$-1.3343\times 10^{-4}$	$-2.4\times 10^{-7}$
$\delta \phi_{1c}$	$-3.542\times 10^{-5}$	$-3.575\times 10^{-5}$	$-3.3\times 10^{-7}$
$\delta \phi_{2s}$	$3.830\times 10^{-5}$	$3.848\times 10^{-5}$	$1.8\times 10^{-7}$
$\delta \phi_{2c}$	$-9.28\times 10^{-6}$	$-9.47\times 10^{-6}$	$-1.9\times 10^{-7}$
${\delta \theta }_{1s}$	$-5.521\times 10^{-5}$	$-5.536\times 10^{-5}$	$-1.5\times 10^{-7}$
${\delta \theta }_{1c}$	$1.3629\times 10^{-4}$	$1.3695\times 10^{-4}$	$6.6\times 10^{-7}$
${\delta \theta }_{2s}$	$-5.97\times 10^{-6}$	$-5.76\times 10^{-6}$	$2.1\times 10^{-7}$
${\delta \theta }_{2c}$	$-2.462\times 10^{-5}$	$-2.484\times 10^{-5}$	$-2.2\times 10^{-7}$

**Table 5:** Maximums of navigation errors.

Navigation Errors	Before Calibration	Two-Stage Calibration	Improvement after Two-Stage Calibration	Three-Stage Calibration	Improvement after Three-Stage Calibration
Longitude	0.84	0.58	30.95%	0.33	60.71%
Latitude	0.86	0.60	30.23%	0.26	69.77%
Northern Velocity	0.85	0.70	17.65%	0.56	34.12%
Eastern Velocity	0.87	0.78	10.34%	0.67	22.99%
Roll	0.89	0.50	43.82%	0.38	57.30%
Pitch	0.95	0.58	38.95%	0.43	54.74%
Yaw	0.80	0.54	32.50%	0.44	45.00%
